# Anticancer activities against cholangiocarcinoma, toxicity and pharmacological activities of Thai medicinal plants in animal models

**DOI:** 10.1186/1472-6882-12-23

**Published:** 2012-03-27

**Authors:** Tullayakorn Plengsuriyakarn, Vithoon Viyanant, Veerachai Eursitthichai, Porntipa Picha, Piengchai Kupradinun, Arunporn Itharat, Kesara Na-Bangchang

**Affiliations:** 1 Thailand Center of Excellence for Drug Discovery and Development (TCEDDD), Thammasat University, Pathumthani, Thailand; 2 Department of Research, National Cancer Institute, Ministry of Public Health, Bangkok, Thailand; 3 Applied Thai Traditional Medicine Center, Faculty of Medicine, Thammasat University, Pathumthani, Thailand; 4 Thailand Center of Excellence for Drug Discovery and Development (TCEDDD), Thammasat University, 99 Moo 18 Paholyothin Rd., Klongluang, Pathumthani 12121, Thailand

**Keywords:** Cholangiocarcinoma, Anticancer, Thai medicinal plants, Nude mouse xenograft model

## Abstract

**Background:**

Chemotherapy of cholangiocarcinoma (CCA), a devastating cancer with increasing worldwide incidence and mortality rates, is largely ineffective. The discovery and development of effective chemotherapeutics is urgently needed.

**Methods/Design:**

The study aimed at evaluating anticancer activities, toxicity, and pharmacological activities of the curcumin compound (CUR), the crude ethanolic extracts of rhizomes of *Zingiber officinale *Roscoe (Ginger: ZO) and *Atractylodes lancea *thung. DC (Khod-Kha-Mao: AL), fruits of *Piper chaba *Hunt. (De-Plee: PC), and Pra-Sa-Prao-Yhai formulation (a mixture of parts of 18 Thai medicinal plants: PPF) were investigated in animal models. Anti-cholangiocarcinoma (anti-CCA) was assessed using CCA-xenograft nude mouse model. The antihypertensive, analgesic, anti-inflammatory, antipyretic, and anti-ulcer activities and effects on motor coordination were investigated using Rota-rod test, CODA tail-cuff system, writhing and hot plate tests, carrageenan-induced paw edema test, brewer's yeast test, and alcohol-induced gastric ulcer test, respectively. Acute and subacute toxicity tests were performed according to the OECD guideline for testing of chemicals with modification.

**Results:**

Promising anticancer activity against CCA in nude mouse xenograft model was shown for the ethanolic extract of AL at all oral dose levels (1000, 3000, and 5000 mg/kg body weight) as well as the extracts of ZO, PPF, and CUR compound at the highest dose level (5000, 4000, and 5000 mg/kg body weight, respectively). PC produced no significant anti-CCA activity. Results from acute and subacute toxicity tests both in mice and rats indicate safety profiles of all the test materials in a broad range of dose levels. No significant toxicity except stomach irritation and general CNS depressant signs were observed. Investigation of pharmacological activities of the test materials revealed promising anti-inflammatory (ZO, PPF, and AL), analgesic (CUR and PPF), antipyretic (CUR and AL), antihypertensive (ZO and AL), and anti-ulcer (CUR, ZO, and AL) activities.

**Conclusion:**

Plants used in Thai traditional medicine for the treatment of various ailments may provide reservoirs of promising candidate chemotherapeutics for the treatment of CCA.

## Background

Cholangiocarcinoma (CCA) is a devastating cancer with increasing worldwide incidence and mortality rate. It is an important public health problem in several parts of Southeast Asia, particularly in the northeastern region of Thailand. The major cause of CCA in Thailand is the consumption of improperly cooked and fermented fresh water cyprinoids fish called "Pla-ra" or "Pla-som", which contains *Opisthorchis viverrini *(OV) and nitrosamine. Lack of effective diagnostic tool and chemotherapeutics are the major constraints for controlling this type of cancer. Chemotherapy of CCA is largely ineffective and clinical efficacy of the standard treatment with 5-fluorouracil (5-FU) is low. Furthermore, the resistance of CCA to chemotherapy and radiotherapy is a major problem [1]. The discovery and development of chemotherapeutics that are effective for the treatment and control of CCA is urgently needed.

Throughout history, natural products have afforded a rich repository of remedies with diverse chemical structures and bioactivities against several heath disorders including cancer. It is estimated that 122 drugs from 94 plant species have been discovered through ethnobotanical leads [2]. Additionally, the use of herbs as complementary and alternative medicine has increased dramatically in the last 20-25 years. According to the World Health Organization (WHO), traditional medicines are relied upon by 65-80% of the world's population for their primary health care needs [2]. In a previous study [3], we screened a total of 28 plants and 5 herbal formulations used in Thai traditional medicine for the treatment of various ailments for their cytotoxic activities against human CCA cell line CL-6. Ethanolic extracts of 5 plants (rhizomes of *Zingiber officinale *Roscoe and *Atractylodes lancea *thung. DC, fruits of *Piper chaba *Hunt., flowers of *Mesua ferrea*, and leaves of *Kaemferia galangal*) and 1 herbal formulation (Pra-Sa-Prao-Yhai) showed promising activities against CL-6 cell line with IC_50 _(concentration that inhibits cell growth by 50%) values of less than 50 μg/ml.

In our effort to search for new herbal medicines with promising activity against CCA, we further investigated the *in vivo *anti-CCA, including the pharmacological activities (motor coordination, antihypertension, analgesia, anti-inflammation, anti-ulcer, and anti-pyrexia) of the ethanolic extracts of rhizomes of *Zingiber officinal *Roscoe (ZO), *Atractylodes lancea *thung. DC (AL), fruits of *Piper chaba *Hunt. (PC), and Pra-Sa-Prao-Yhai formulation (PPF) in CCA-xenograft nude mouse model. Due to the potential therapeutic interest for the treatment of cancer, the anti-CCA and pharmacological activities of curcumin (CUR), the phenolic compound isolated from rhizome of *Curcuma longa *Linn. was also investigated. ZO, commonly known as ginger, is a familiar condiment for various foods and beverages and is used in folk medicine in Asia and in tropical areas for various purposes such as relief for colds, fevers, and digestive problems and as treatment for nausea and vomiting as well as for arthritis. Some pungent constituents present in ZO possess potent antioxidant and anti-inflammatory activities and some of them exhibit cancer preventive activity in experimental carcinogenesis [4]. AL, the dried rhizome of *A. lancea *thung. DC called "Cang Zhu" in China or "Khod-Kha-Mao" in Thailand, has traditionally been used as a stomachic in China and Japan [5]. PC, the fruit of *P. chaba *Hunt. commonly called "Dee Plee", has been used in Thai medicine as an anti-flatulent, expectorant, carminative, antitussive, antifungal, uterus contracting agent, sedative-hypnotic, appetizer, counter-irritant, and is also useful in asthma, bronchitis, fever, and inflammation [6]. PPF is a Thai traditional medicine used for the treatment of fever in children [7]. This remedy consists of a mixture of various parts of 18 medicinal plants as described in Table 1.

**Table 1:** Medicinal plants and herbal formulations under investigation

Family	Plant	Part used	Voucher specimen	Thai traditional Use
Zingiberaceae	*Zingiber officinale* Roscoe.	Rh	SKP 206261501	Treatment of hypercholesteremia and high level triglyceride

Compositae	*Atractylodes lancea *(Thung.) DC.	Rh	SKP 051011201	Treatment of fever, colds, flu, sore throat

Piperaceae	*Piper chaba *Hunt.	Fr	SKP 146160301	Used as carminative, antidiarrheal

**Composition of Pra-Sa Prao-Yhai formulation:**				

Compositae	*Artemisia annua* Linn.	Rh	SKP 051010101	Treatment of fever, hemorrhoids

Compositae	*Atractylodes lancea *(Thung.) DC.	Rh	SKP 051011201	Treatment of fever, colds, flu, sore throat

Cruciferae	*Asclepias curassavica* Linn.	Fl	SKP 057121901	Used as analgesic

Dracaenaceae	*Dracaena loureiri* Gagnep.	St, Ba	SKP 065041201	Treatment of cough, fever, inflammation

Guttiferae	*Mammea siamensis *Kosterm.	Fl	SKP 083131901	Restorative

Guttiferae	*Mesua ferrea *Linn.	Fl	SKP 083130601	Treatment of dyspepsia

Myristicaceae	*Myristica fragrans *Houtt.	Sd	SKP 121130601	Treatment of uterus pain, diarrhea

Myrtaceae	*Syzgium aromaticum *(L.) Merr. & L.M. Perry	Fl	SKP 123190101	Treatment of toothache, bacterial infection

Nelumbonaceae	*Nigella sativa *Linn.	Sd	SKP 160141901	Treatment of jaundice

Sapotadeae	*Mimusops elengi *Linn.	Fl	SKP 171130501	Used as cordial, tonic. Treatment of syncope

Umbelliferae	*Angelica dahurica *Benth.	Rt	SKP 199010401	Used as antipyretic, antiasthma, anticough

Umbelliferae	*Angelica sinensis *(Oliv.) Diels	Rh	SKP 199010901	Treatment of bronchitis pleurisy

Umbelliferae	*Anethum graveolens *Linn.	Rt, Fr	SKP 199010701	Used as carminative. Treatment of eye pain

Umbelliferae	*Cuminum cyminum *Linn.	Sd	SKP 199030301	Treatment of dyspepsia, diarrhoea and jaundice

Umbelliferae	*Foeniculum vulgare *Mill. var. dulce Alef.	Sd	SKP 199062201	Used as analeptic

Umbelliferae	*Ligusticum sinense *Oliv. cv. Chuanxiong	Rh	SKP 199121901	Treatment of urinary bladder channel,, headache, neurodermatitis

Zingiberaceae	*Amomum testaceum *Ridl.	Sd	SKP 206011101	Used as carminative, antibacterial

Zingiberaceae	*Kaempferia galangal*	Lf	SKP 206110701	Antinociceptive, anti-inflammatory

## Results

### Toxicity study

For CUR, ZO, and AL, the maximum tolerated, including the medium and low dose levels that produced no significant sign of toxicity or death in the acute and sub-acute toxicity tests, were 5,000, 3,000, and 1,000 mg/kg body weight, respectively. The maximum tolerated, medium and low dose levels of PPF, were 4,000, 2,000, and 1,000 mg/kg body weight. The corresponding dose levels for PC were 1,000, 500, and 100 mg/kg body weight, respectively. In all these cases, no significant toxicity, except stomach irritation and general CNS depressant signs (reduced alertness and locomotion and diminished response to touch and balance) was observed. Stomach irritation was observed in all animals immediately after feeding them with the highest dose of test materials but the symptom subsided within 2 hours of administration.

### Anti-cholangiocarcinoma activity

#### Tumor volume

**Figure 1:**
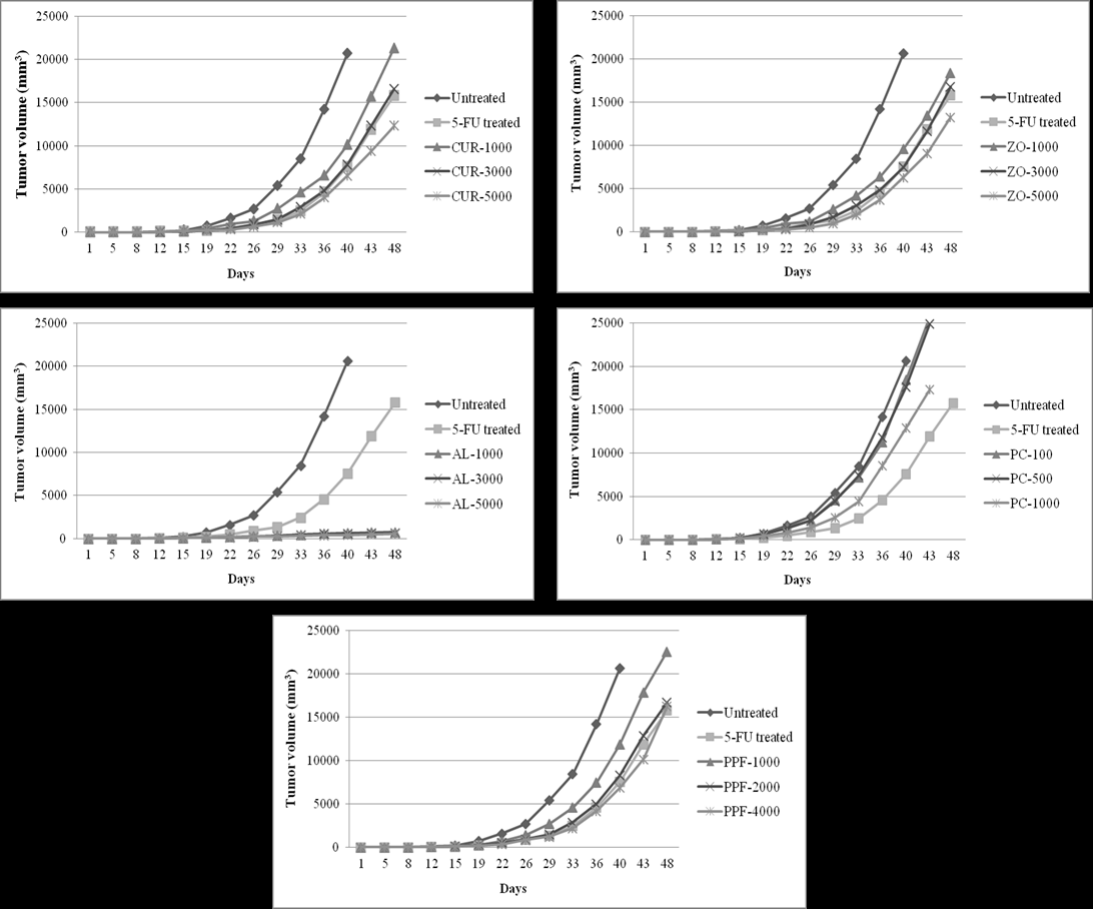
**Anti-CCA activities indicated by inhibitory action on tumor volume (mean ± SEM: mm^3^) of CUR compound and extracts of ZO, AL, PC, and PPF at various dose levels, in comparison with NSS (untreated control) and 5-FU (reference control) in CCA-xenografted nude mice during the 48 days investigation period**. Statistically significant difference by ANOVA, followed by post hoc scheffe test: AL-treated group at low dose level vs. control group (p < 0.001). AL-treated group at medium dose level vs. control group (p < 0.001). AL-treated group at high dose level vs. control group (p < 0.001). CUR-treated group at high dose level vs. control group (p < 0.01). ZO-treated group at high dose level vs. control group (p < 0.01). PPF-treated group at high dose level vs. control group (p < 0.01). PC-treated group at high dose level vs. control group (p < 0.01)

**Figure 2:**
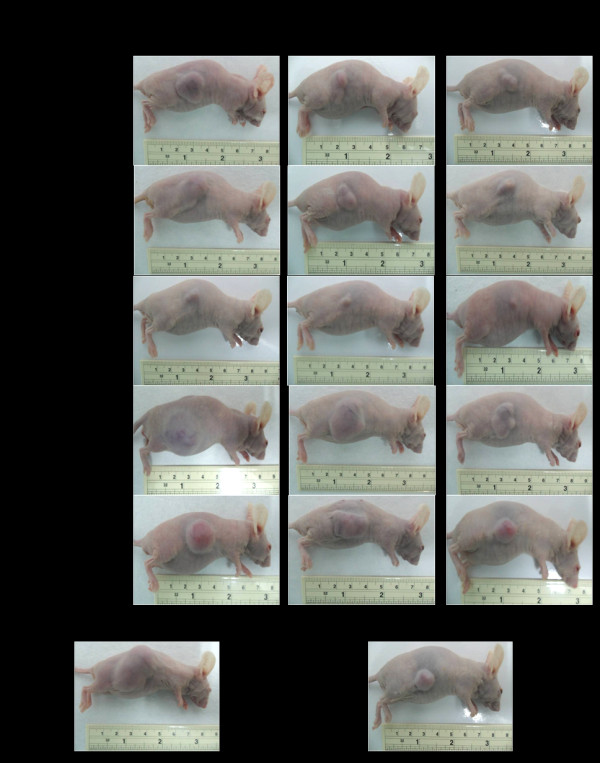
**Representative tumors of CCA-xenografted nude mice following treatment with the test materials (CUR compound and extracts of ZO, AL, PC, and PPF at various dose levels) in comparison with untreated control and 5-FU (reference control)**.

To assess the inhibitory activities of the test materials (CUR compound and plant extracts) on tumor growth, CL-6 cells were injected subcutaneously into the lower flanks of the CCA-xenografted nude mice. Tumor growth inhibition was most evident in the mice treated with AL at all dose levels, of which less than 10% of the tumor size of the control group was observed on day 40 (Figure 1). The highest dose level (5,000 mg/kg body weight) of CUR, ZO, PC, and PPF exhibited low activities on day 48 (40.5, 35.8, 16.1, and 21.2% tumor volume of the control group, respectively). Mean (± SEM) of tumor volumes for the control and the groups treated with 5-FU, CUR, ZO, AL, PC, and PPF were 20,661 ± 126, 15,789 ± 101, 12,290 ± 144, 13,270 ± 130, 550 ± 13, 17,347 ± 116, and 16,290 ± 116 mm^3^, respectively. Representative tumors of CCA-xenografted mice following administration of test materials are shown in Figure 2.

#### Survival time

**Figure 3:**
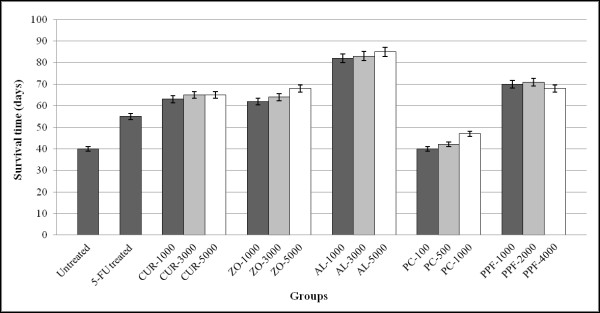
**Anti-CCA activities indicated by prolongation of survival time (mean ± SEM), of CUR compound and extracts of ZO, AL, PC, and PPF at various dose levels, in comparison with NSS (untreated control) and 5-FU (reference control) in CCA-xenografted nude mice during the 85 days investigation period**. Statistically significant difference by ANOVA, followed by post hoc scheffe test: AL-treated group at low dose level vs. control group (p < 0.001). AL-treated group at medium dose level vs. control group (p < 0.001). AL-treated group at high dose level vs. control group (p < 0.001).

CCA-xenografted nude mice receiving AL at all dose levels had a significant (*p *< 0.001) prolongation of survival time (mean ± SEM) over 80 days (83.3 ± 0.88 days) after tumor transplantation compared with the untreated group (40.0 ± 0.57 days). The survival time of mice receiving 5-FU, CUR, ZO, PC, and PPF were 55.0 ± 0.87, 64.3 ± 0.67, 64.7 ± 1.76, 43.0 ± 2.08, and 69.7 ± 0.88 days, respectively (Figure 3).

#### Histopathology

**Figure 4:**
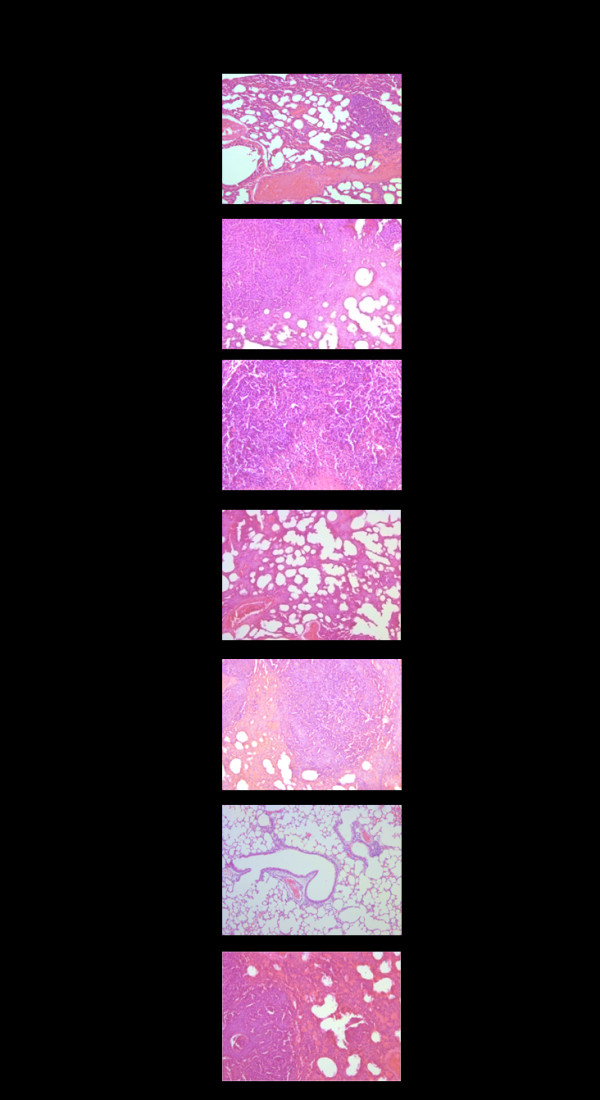
**Representative histopathology of lung metastasis of CCA-xenografted nude mice following treatment with the test materials (CUR compound and extracts of ZO, AL, PC, and PPF at various dose levels) in comparison with untreated control and 5-FU (reference control)**. % Metastasis of lung mass represents mean (± SEM) from 6 mice for each group.

Histopathological findings revealed prominent inhibition of lung metastasis by AL at the highest dose of 5,000 mg/kg body weight. Lung metastasis occurred in both the untreated and treated mice but the intensity of lung metastasis was significantly lower following a high dose AL, with an average of less than 5% of the total lung mass. In contrast, the metastasis covered about 90% of the total lung mass of the untreated mice (Figure 4). Mice receiving 5-FU, CUR, PC, and PPF developed lung metastasis of 50, 60, 50, 80, and 40% of the total lung mass, respectively.

### Pharmacological activities

#### Motor coordination activity

**Figure 5:**
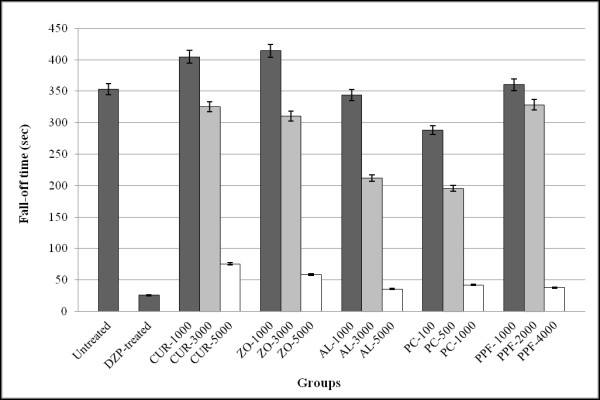
**Effect of test materials on locomotive activity (represented by mean ± SEM of fall-off time in seconds) in rats following treatment with the test materials (CUR compound and extracts of ZO, AL, PC, and PPF at various dose levels) in comparison with untreated control and diazepam (reference control)**. Statistically significant difference by ANOVA, followed by post hoc scheffe test: CUR-treated group at high dose level vs. control group (p < 0.001). ZO-treated group at high dose level vs. control group (p < 0.001). AL-treated group at high dose level vs. control group (p < 0.001). PC-treated group at high dose vs. control group (p < 0.001) PPF-treated group at high dose vs. control group (p < 0.001). Diazepam-treated group vs. control group (p < 0.001).

Compared with the untreated group, a high dose level of CUR, ZO, AL, PC, and PPF produced a significant (*p *< 0.001) interference with muscle relaxation similar to that produced by 4 mg/kg body weight diazepam (Figure 5). The mean (± SEM) fall off time in the untreated and the groups treated with diazepam, high dose level of CUR, ZO, AL, PC, and PPF were at 353.3 ± 11.5, 25.5 ± 1.8, 75.6 ± 2.9, 58.4 ± 2.4, 35.4 ± 2.5, 41.9 ± 2.7, and 37.8 ± 2.6 sec, respectively.

#### Antihypertensive activity

High dose CUR, AL, PC, and the reference drug propanolol (10 mg/kg body weight) significantly (*p *< 0.001) decreased systolic (sBP) and diastolic (dBP) blood pressure of rats following treatment with adrenaline (Table 2). Propanolol (10 mg/kg body weight) and all doses of AL significantly (*p *< 0.001) reduced the heart rate of rats compared with the baseline. For PC, only medium (500 mg/kg body weight) and high (1,000 mg/kg body weight) dose levels significantly (*p *< 0.001) decreased heart rate. PPF and CUR had no effect on heart rate reduction (Table 3). The effect of all test materials on arterial blood pressure was similar to that observed with the heart rate (Table 4).

**Table 2:** Effect of test materials and propanolol on systolic (sBP) and diastolic (dBP) blood pressure of rats

Group	Dose (mg/kg bw.)	Blood Pressure (mmHg)					
		
		Before induction of hypertension		After induction of hypertension		After treatment of hypertension	
		
		sBP	dBP	sBP	dBP	sBP	dBP
**Normal**	-	118 ± 0.4	81 ± 0.3	120 ± 0.3	82 ± 0.4	120 ± 0.3*	81 ± 0.4*

**Untreated control**	-	119 ± 0.5	80 ± 0.4	178 ± 0.4	128 ± 0.4	181 ± 0.6	137* ± 0.4

**Propanolol-treated**	10	120 ± 0.6	81 ± 0.3	177 ± 0.4	131 ± 0.5	135 ± 0.4*	95 ± 0.4*

**CUR**	1000	119 ± 0.5	82 ± 0.3	178 ± 0.3	130 ± 0.4	164 ± 0.4	126 ± 0.3
	
	3000	119 ± 0.4	79 ± 0.4	180 ± 0.4	131 ± 0.5	152 ± 0.3	121 ± 0.4
	
	5000	118 ± 0.4	78 ± 0.3	179 ± 0.4	130 ± 0.4	136 ± 0.4*	101 ± 0.4*

**ZO**	1000	119 ± 0.5	81 ± 0.3	178 ± 0.5	130 ± 0.5	168 ± 0.3	119 ± 0.3
	
	3000	120 ± 0.5	80 ± 0.3	180 ± 0.4	129 ± 0.5	163 ± 0.5	120 ± 0.5
	
	5000	120 ± 0.4	81 ± 0.4	181 ± 0.5	128 ± 0.4	162 ± 0.4	119 ± 0.4

**AL**	1000	121 ± 0.5	79 ± 0.4	180 ± 0.4	131 ± 0.5	155 ± 0.3	109 ± 0.4
	
	3000	118 ± 0.4	82 ± 0.4	179 ± 0.5	126 ± 0.5	156 ± 0.4	105 ± 0.5
	
	5000	119 ± 0.3	81 ± 0.3	177 ± 0.4	169 ± 0.4	134 ± 0.4*	98 ± 0.3*

**PC**	100	117 ± 0.3	79 ± 0.4	180 ± 0.4	127 ± 0.5	180 ± 0.5	126 ± 0.4
	
	500	118 ± 0.4	81 ± 0.3	177 ± 0.3	129 ± 0.4	170 ± 0.4	122 ± 0.4
	
	1000	120 ± 0.5	80 ± 0.4	179 ± 0.4	128 ± 0.4	139 ± 0.3*	113 ± 0.4*

**PPF**	1000	119 ± 0.4	82 ± 0.4	179 ± 0.4	131 ± 0.4	169 ± 0.5	117 ± 0.3
	
	3000	120 ± 0.5	79 ± 0.4	180 ± 0.5	130 ± 3.3	162 ± 0.4	121 ± 0.4
	
	4000	119 ± 0.4	80 ± 0.3	181 ± 0.5	128 ± 0.5	162 ± 0.4	120 ± 0.5

Data are presented as mean ± SEM values

*Statistically significant difference with baseline by paired t-test:

sBP and dBP of CUR-treated group at high dose level (p < 0.001)

sBP and dBP of AL-treated group at high dose level (p < 0.001)

sBP and dBP of PC-treated group at high dose level (p < 0.001)

sBP and dBP of propanolol-treated group at high dose level (p < 0.001)

**Table 3:** Effect of test materials and propanolol (reference control) on heart rate of rats

Group	Dose (mg/kg bw.)	Heart rate (beat/min)		
		
		Before induction of hypertension	After induction of hypertension	After treatment of hypertension
**Normal**	-	412 ± 4.3	415 ± 4.1	409 ± 4.5

**Untreated control**	-	399 ± 4.1	535 ± 5.4	570 ± 5.8

**Propanolol-treated**	10	410 ± 4.4	520 ± 5.5	405 ± 4.4

**CUR**	1000	411 ± 4.5	530 ± 5.5	530 ± 5.5
	
	3000	401 ± 4.2	528 ± 5.3	518 ± 5.6
	
	5000	390 ± 3.9	523 ± 5.4	471 ± 4.3

**ZO**	1000	420 ± 4.2	526 ± 4.9	499 ± 5.1
	
	3000	418 ± 4.9	531 ± 5.7	474 ± 5.2
	
	5000	415 ± 4.6	530 ± 5.5	416 ± 4.1*

**AL**	1000	403 ± 4.1	544 ± 5.4	431 ± 4.4*
	
	3000	407 ± 4.3	540 ± 5.6	416 ± 4.3*
	
	5000	398 ± 3.6	528 ± 5.3	410 ± 4.5*

**PC**	100	409 ± 4.0	537 ± 5.2	540 ± 5.6
	
	500	410 ± 4.2	519 ± 5.3	427 ± 4.1*
	
	1000	420 ± 4.5	509 ± 5.3	419 ± 4.6*

**PPF**	1000	412 ± 4.5	519 ± 5.2	559 ± 5.8
	
	2000	398 ± 4.7	534 ± 5.6	544 ± 5.7
	
	4000	405 ± 4.4	526 ± 5.5	536 ± 5.4

Data are presented as mean ± SEM values

*Statistically significant difference with baseline by paired t-test:

sBP and dBP of ZO-treated group at high dose level (p < 0.001)

sBP and dBP of AL-treated group at all dose levels (p < 0.001)

sBP and dBP of PC-treated group at medium and high dose levels (p < 0.001)

**Table 4:** Effect of test materials and propanolol (reference control) on mean arterial blood pressure of rats

Group	Dose (mg/kg bw.)	Mean arterial pressure (mmHg)		
		
		Before induction of hypertension	After induction of hypertension	After treatment of hypertension
**Normal**	-	107 ± 3.1	110 ± 3.4	109 ± 2.7

**Untreated control**	-	108 ± 3.4	143 ± 2.7	151 ± 3.0

**Propanolol-treated**	10	110 ± 3.2	145 ± 3.1	116 ± 2.5*

**CUR**	1000	106 ± 0.4	145 ± 0.3	138 ± 0.5
	
	3000	104 ± 0.3	146 ± 0.4	137 ± 0.4
	
	5000	107 ± 0.3	148 ± 0.3	135 ± 0.4

**ZO**	1000	105 ± 0.3	144 ± 0.4	135 ± 0.5
	
	3000	107 ± 0.4	147 ± 0.5	137 ± 0.3
	
	5000	106 ± 0.3	148 ± 0.4	119 ± 0.3*

**AL**	1000	105 ± 0.3	148 ± 0.5	118 ± 0.4*
	
	3000	107 ± 0.4	144 ± 0.4	120 ± 0.4*
	
	5000	106 ± 0.3	148 ± 0.4	122 ± 0.3*

**PC**	100	103 ± 0.3	147 ± 0.3	134 ± 0.5
	
	500	105 ± 0.4	149 ± 0.4	124 ± 0.4*
	
	1000	105 ± 0.4	147 ± 0.4	125 ± 0.4*

**PPF**	1000	104 ± 0.3	145 ± 0.4	134 ± 0.3
	
	2000	108 ± 0.4	146 ± 0.5	134 ± 0.4
	
	4000	104 ± 0.4	143 ± 0.5	134 ± 0.5

Data are presented as mean ± SEM values

*Statistically significant difference with baseline by paired t-test:

sBP and dBP of ZO-treated group at high dose level (p < 0.001)

sBP and dBP of AL-treated group at all dose levels (p < 0.001)

sBP and dBP of PC-treated group at medium and high dose levels (p < 0.001)

sBP and dBP of propanolol-treated group (p < 0.001)

#### Analgesic activity

**Figure 6:**
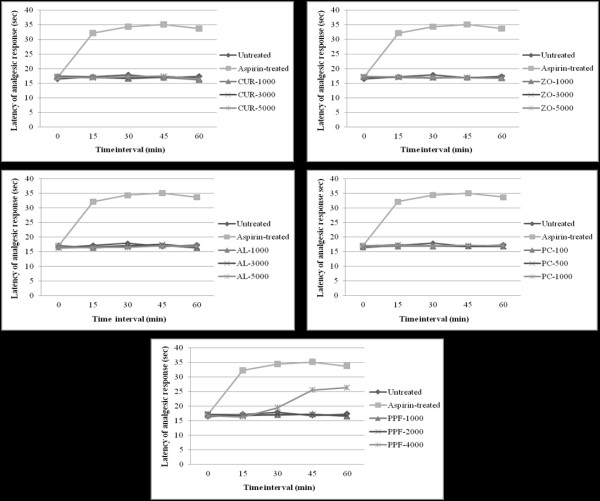
**Central analgesic activity (represented by mean ± SEM of analgesic response in hot plate test) of CUR compound and extracts of ZO, AL, PC, and PPF at various dose levels, in comparison with untreated control and aspirin (reference control)**. Statistically significant difference by ANOVA, followed by post hoc scheffe test: PPF-treated group at high dose level vs. control group (p < 0.01). Aspirin-treated group vs. control group (p < 0.001).

The central analgesic activities (hot plate test) of the test materials were supported by the results of the hot plate tests. Oral administration of a high dose level of PPF produced a significant (*p *< 0.001) prolongation of the latency time to the heat stimulus (with potency of about 50% of 5 mg/kg body weight morphine) compared with the untreated control, starting 30 min until 60 min after administration. CUR, AL, and PC did not produce any significant central analgesic activities (Figure 6).

**Figure 7:**
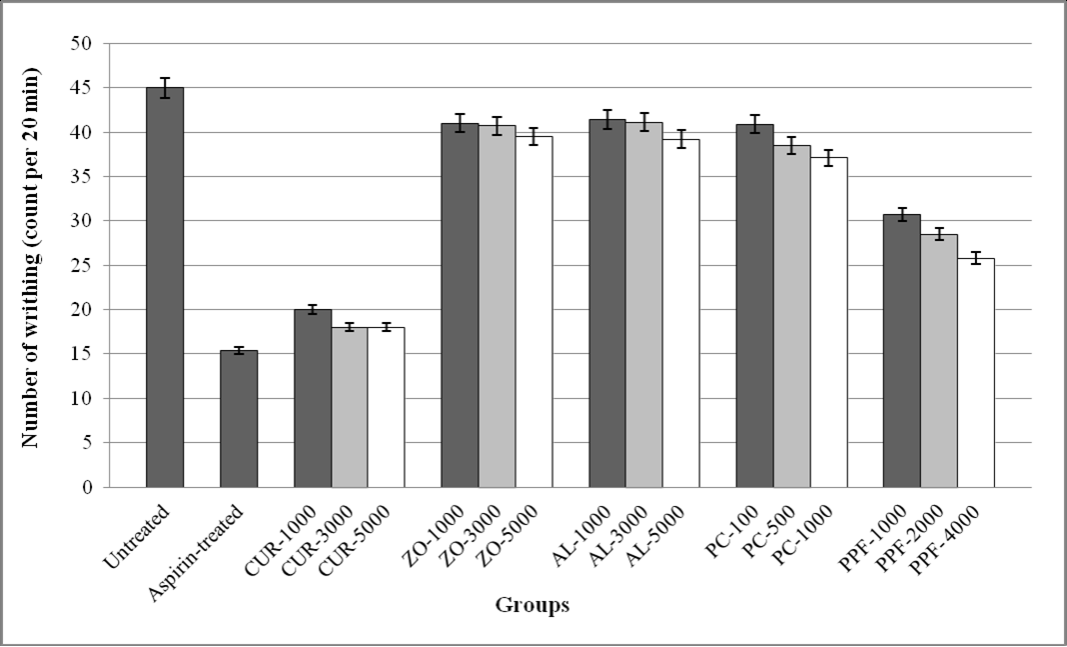
**Peripheral analgesic activity (represented by mean ± SEM) number of writhing and stretching in writhing test) of CUR compound and extracts of ZO, AL, PC, and PPF at various dose levels, in comparison with untreated control and aspirin (reference control)**. Statistically significant difference by ANOVA, followed by post hoc scheffe test: CUR-treated group at low dose level vs. control group (p < 0.01). CUR-treated group at medium dose level vs. control group (p < 0.001). CUR-treated group at high dose level vs. control group (p < 0.001). Aspirin-treated group vs. control group (p < 0.001).

For peripheral analgesic activity (Writhing test), oral administration of CUR compound at all dose levels significantly (*p *< 0.01) inhibited the number of writhings and stretching induced by 0.6% acetic acid given by intraperitoneal injection with a potency of about 70-80% of aspirin (200 mg/kg body weight). No significant peripheral analgesic activity was observed with AL, PC, and PPF (Figure 7).

#### Anti-inflammatory activity

**Figure 8:**
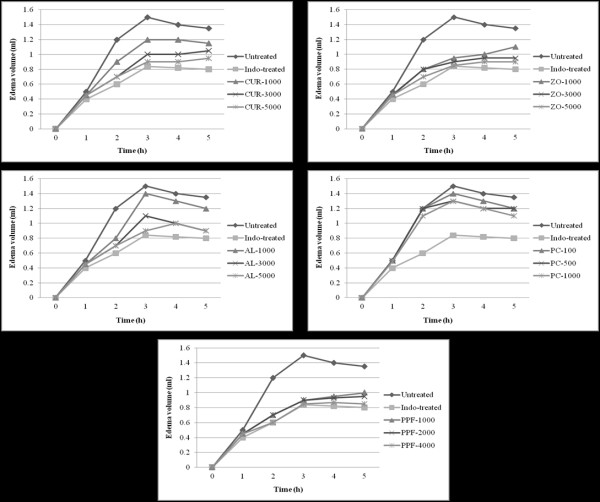
**Inflammatory activity (represented by mean ± SEM change of edema volume in λ-carrageenan-induced paw edema test) of CUR compound and extracts of ZO, AL, PC, and PPF at various dose levels, in comparison with untreated control and indomethacin (reference control)**. Statistically significant difference by ANOVA, followed by post hoc scheffe test: CUR-treated group at low dose level vs. control group (p < 0.01). CUR-treated group at medium dose level vs. control group (p < 0.01). CUR-treated group at high dose level vs. control group (p < 0.01). Aspirin-treated group vs. control group (p < 0.001).

Compared with the untreated control, indomethacin at a dose of 10 mg/kg body weight significantly (*p *< 0.001) reduced the paw edema volume induced by carrageenan, starting three hours after administration. Similarly, PPF at all dose levels also produced a significant (*p *< 0.01) anti-inflammatory activity. AL exhibited significant (*p *< 0.01) activity only at a high dose level whereas CUR exhibited significant activity at medium and high dose levels (Figure 8).

#### Anti-ulcer activity

**Figure 9:**
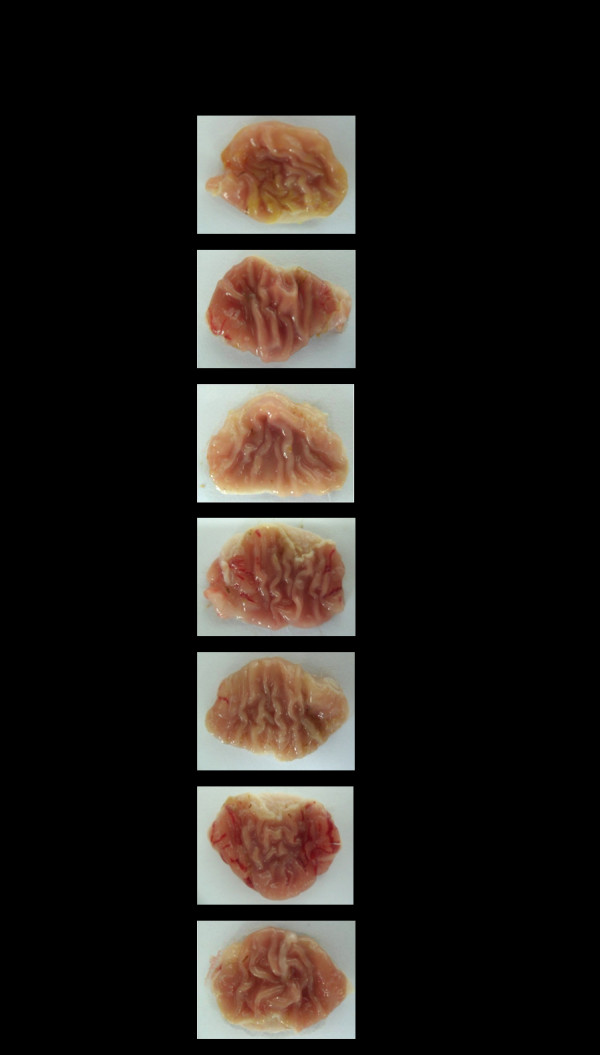
**Observed ulcer area in gross appearance and inhibition percentage in rats**.

**Figure 10:**
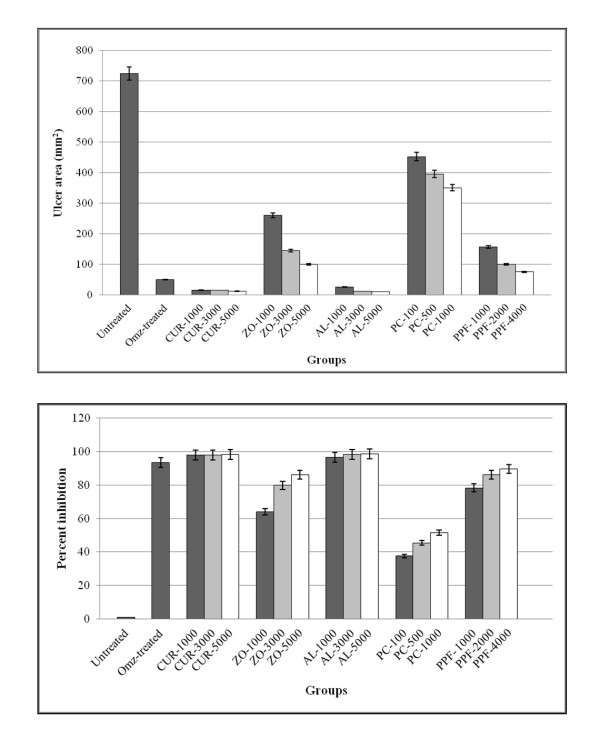
**Anti-ulcer activity (represented by mean ± SEM of ulcer area and % inhibition of ulcer area in ethanol-induced gastric ulcer test) of CUR compound and extracts of ZO, AL, PC, and PPF at various dose levels, in comparison with untreated control and omeprazole (reference control)**. Statistically significant difference by ANOVA, followed by post hoc scheffe test: CUR-treated group at low dose level vs. control group (p < 0.001). ZO-treated group at medium dose level vs. control group (p < 0.001). AL-treated group at high dose level vs. control group (p < 0.001). PPF-treated group at high dose level vs. control group (p < 0.001). Omeprazole-treated group vs. control group (p < 0.001).

The anti-ulcer activities of all test materials on ethanol-induced gastric lesion model are shown in Figure 9. Significant reduction (96.47-98.24%) of ulcer size was observed in rats pre-treated with AL (*p *< 0.001) and CUR (*p *< 0.001) at all dose levels compared to the untreated control. Their potencies of activity were comparable to the reference drug omeprazole given at a dose of 20 mg/kg body weight (93.04%) (Figure 10).

#### Antipyretic activity

**Figure 11:**
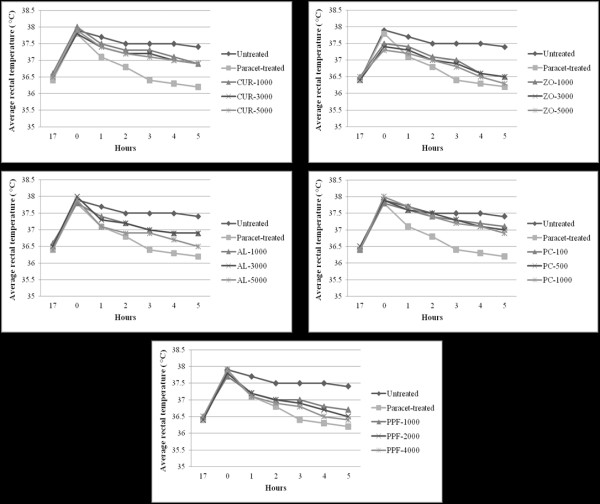
**Antipyretic activity (represented by mean ± SEM of rectal temperature in brewer's yeast induced pyrexia test) of CUR compound and extracts of ZO, AL, PC, and PPF at various dose levels, in comparison with untreated control and omeprazole (reference control)**. Statistically significant difference by ANOVA, followed by post hoc scheffe test: PPF-treated group at medium dose level vs. control group (p < 0.01). ZO-treated group at high dose level vs. control group (p < 0.01). PC-treated group at high dose level vs. control group (p < 0.01). PPF-treated group at high dose level vs. control group (p < 0.01). Aspirin-treated group vs. control group (p < 0.001).

The experimental rats showed a mean increase in about 1.4°C of rectal temperature, 17 hours after Brewer's yeast injection. PPF at 2,000 mg/kg body weight, and ZO and AL at 5,000 mg/kg body weight produced a significant (*p *< 0.01) antipyretic activity at 5 hours after administration, whereas PPF at 4,000 mg/kg and aspirin at 200 mg/kg body weight showed significant antipyretic activity throughout the observation period starting from 3 up to 6 hours (Figure 11).

## Discussion

The present study demonstrated promising anticancer activity against CCA in nude mouse xenograft model of the ethanolic extract of AL at all dose levels, as well as the extracts of ZO, PPF, and CUR compound at the highest dose level. PC produced no significant anti-CCA activity. Results from the acute and subacute toxicity tests both in mice and rats indicated safety profiles of all test materials in a broad range of dose levels. No significant toxicity, except stomach irritation and general CNS depressant signs (reduced alertness and locomotion, and diminished response to touch and balance), was observed. Stomach irritation occurred in all animals immediately after feeding them with a high dose of the test materials. Investigation of pharmacological activities of the test materials revealed promising anti-inflammatory (ZO, PPF, and AL), analgesic (CUR and PPF), antipyretic (CUR and AL), antihypertensive (ZO and AL), and anti-ulcer (CUR, ZO, and AL) activities.

The ethanolic extract of AL was shown to possess the most potent anti-CCA activity similar to 5-FU with regards to the reduction of tumor mass, prolongation of survival time, and inhibition of lung metastasis. All dose levels significantly reduced tumor size (by 97.3%), prolonged survival time (by 208.5%), and inhibited lung metastasis (by 95% of total lung mass) compared with the untreated control. To the best of our knowledge, the present study is the first study that demonstrated the anticancer activity of AL. Interestingly, AL extract exhibited prominent inhibitory effect on lung metastasis. Metastasis is one of the major problems in the treatment of several cancer types. In severe stage CCA, metastatic tumors in the lungs are CCA cancers which developed in the lung tissues by spreading from the liver origin through the bloodstream or lymphatic system to the lungs. Histopathological examination at autopsy revealed lung metastasis in all mice following xenografting with CL-6. Significant reduction of lung metastasis to only 5% of the total lung mass was observed in the xenografted mice treated with AL (Figure 4) whereas metastasis of more than 90% of the total lung mass was found in the untreated mice. The anti-metastatic action of AL corresponded well with its observed antihypertensive effects (reduction of systolic and diastolic blood pressure, heart rate, and mean arterial pressure). Furthermore, AL also exhibited anti-ulcer (all dose levels), anti-inflammatory (high dose), and antipyretic (high dose) activities. The observed significant anti-ulcer activity (96-98%) of AL confirms its use to improve stomach damage partly through anti-ulcer effects [8]. This anti-ulcer activity was more potent than the reference drug omeprazole given at a dose of 20 mg/kg body weight. The compound 2-[(2'E)-3',7'-dimethyl-2',6'-octadienyl]-4-methoxy-6-methylphenol isolated from the rhizome of AL showed strong inhibitory effects on 5-lipoxygenase (5-LOX) and cyclooxygenase-1 (COX-1) [9], the two key enzymes responsible for the metabolism of arachidonic acid, either to prostaglandins and thromboxanes or to leukotrienes, which play a central role in the regulation of different physiological processes, but also cause pain, inflammation, and hypersensitivity.

Significant anti-CCA activity of rhizome extract of ZO was also observed in the xenograft mouse model. High dose ZO significantly reduced tumor volume (by 35.8%), and all dose levels significantly prolonged survival time (by 161.5%) compared with the control group. Inhibition of lung metastasis by about half was seen at high dose. Furthermore, the extract possessed anti-inflammatory at all dose levels. In our previous study, the anti-CCA activity of ZO was demonstrated in OV/dimethylnitrosamine induced-CCA hamster model [10]. The extract significantly prolonged survival time and survival rate of the cancerous animals. The anticancer properties of ZO have been attributed to the presence of certain pungent vallinoids, *viz*. [6]-gingerol and [6]-paradol, as well as some other constituents like shogaols and zingerone [11]. A number of mechanisms that may be involved in the chemopreventive activity of ZO and its components have been reported from the laboratory studies in various experimental models [11]. Various studies have shown that a wide range of ZO constituents inhibit production of nitric oxide, inflammatory cytokines and enzymes prostaglandin synthase, and arachidonate-5-LOX in a dose-dependent manner. The latter in turn, inhibits the synthesis of leukotrienes from both COX-1 and COX-2 and LOX, respectively [12]. ZO has also demonstrated a significant reduction of inflammation in animals compared with conventional drugs [12]. A significant anti-ulcer activity of the ethanolic extract of ZO was also observed. In a previous study [13], the gastroprotective effect of 50% ethanolic extract of ZO was assessed in rats in the ethanol and acetic acid-induced ulcer models at different doses (500 mg/kg and 1.5-5 g/kg body weight, respectively). The extract showed a dose-dependent inhibition of the ulcer index in ethanol and acetic acid-induced ulcers. It prevented the oxidative damage of the gastric mucosa by blocking lipid peroxidation and by a significant decrease in superoxide dismutase and increase in catalase activity.

The anti-CCA activity of PPF extract was moderate; nevertheless, it exhibited diverse pharmacological activities. High dose PPF significantly reduced tumor volume and prolonged survival time compared with the untreated group. Inhibition of lung metastasis occurred on an average 60% of the lung mass. The anticancer activity of PPF is likely to be due to the activities of various components in the formulation. Recently, we demonstrated the cytotoxic activity against CL-6, a CCA cell line, of the two components of PPF: *Mimusops elengi *Linn. (flower) (IC_50 _= 48.53 μg/ml) and *Kaempferia galangal *("Proh-hom" in Thai; leaf) (IC_50 _= 37.36 μg/ml) [3]. In addition, previous *in vitro *and *in vivo *studies reported the anticancer potential of *Nigella saliva *(commonly known as black seed or black cumin) [14], *Angelica sinensis *(Oliv.) Diels [15], *Anethum graveolens *Linn. [16], *Foeniculum vulgare *Mill. [17], *Angelica dahurica *Benth. [18], *Mammea siamensis Kosterm*. [19], *Myristica fragrans *Houtt. [20], and *Syzygium aromaticum *Linn. (cloves) [21]. The bioactive compound derived from *N. sativa *oil is thymoquinone, which was shown to exhibit anti-tumor activities, including anti-proliferative and pro-apoptotic effects on cell lines derived from breast, colon, ovary, larynx, lung, myeloblastic leukemia, and osteosarcoma [22-26]. Mechanistically, thymoquinone reportedly induced apoptosis in tumor cells by suppressing NF-κB, Akt activation and extracellular signal-regulated kinase signaling pathways, and also inhibits tumor angiogenesis [27]. Recently, the serine/threonine Polo-like kinases (Plk) which are over-expressed in many types of human cancers have been identified as targets for thymoquinone. Cytotoxic activity towards several cell lines *in vitro *including apoptotic activity was also shown with a novel polysaccharide isolated from a rhizome of *A. sinensis *(Oliv.) Diels named APS-1 d [15]. Among the coumarins isolated from the bark of *M. siamensis *Kosterm, theraphin C showed the strongest inhibitory activity on cell proliferation in DLD-1 (colon cancer), MCF-7 (breast adenocarcinoma), HeLa (human cervical cancer), and NCI-H460 (human lung cancer) cell lines [19].

With regards to the pharmacological activities of PPF, anti-inflammatory and CNS suppression effects were observed at all doses, whereas reduction of blood pressure and analgesic effects were shown at high dose and antipyretic effect was shown at the medium dose level of 2,000 mg/kg body weight. Promising antipyretic, anti-hypertensive, anti-inflammatory, and analgesic activities of various components of PPF have previously been shown. For example, the seed of *N. sativa *was shown to exhibit antihypertensive property [14]. The anti-inflammatory action of nutmeg has been shown to be due to the myristicin that it contains [28]. Anti-inflammatory effect of thymoquinone from the seed of *N. sativa *was shown to be due to the inhibitory effect on eicosanoid generation, namely thromboxane B2 and leukotrienes B4, by inhibiting both COX and LOX enzymes, respectively [29]. The ethanol fractions of *A. sinensis *(Oliv.) Diels and *A. dahurica *were shown to exert an anti-inflammatory effect through the suppression of NF-κB-dependent activity [30]. Flavonoids and stilbenoids isolated from the stem wood of *D. loureiri *were shown to exhibit COX-1 and COX-2 inhibitory activity [31]. The anti-inflammatory mechanisms of byakangelicin, the active component of *A. dahurica *Benth, involve the inhibition of tumour necrosis factor-α, histamine release, and of PGE2 through decreased COX-2, besides its potent antioxidant effects [32]. The analgesic activity of PPF observed in the present study was found to be centrally acting. The antinociceptive effects of *N. sativa *oil and the active compound thymoquinone were shown to be through indirect activation of the superspinal mu_1 _and kappa opioid receptors [33].

Recent studies have substantiated and provided scientific evidence regarding the prophylactic and therapeutic potential of CUR as anticancer in several types of cancer including colon, lung, breast, liver, and prostate. Interestingly, the anticancer activity of CUR against CCA in the hamster model was reported recently [34]. The compound exhibited an anticarcinogenic potential *via *increasing the survival of hamsters, suppression of the various events involved in multiple steps of carcinogenesis such as transcription factor, NF-κB, AP-1, and STAT-3, and ability to suppress pro-inflammatory pathways on COX-2 and iNOS. In the present study, the CUR compound was shown to possess moderate anti-CCA activity. High dose significantly reduced tumor volume (by 40.5%) and inhibited lung metastasis (by 60%). All doses significantly prolonged survival time (by 160.8%) compared with the control group. Undisputed scientific evidence suggests that CUR suppresses all three stages of carcinogenesis: initiation, promotion, and progression [34]. Due to a vast number of biological targets with virtually no side effects, CUR has achieved the potential therapeutic interest to cure immune related, metabolic diseases, and cancer. The intricate mechanism of action of CUR involves various biological targets *viz *transcription factors: NF-AT, AP-1, signal transducers and activator of transcription (STAT), p53 and kinases: mitogen-activated protein kinases, cytokines release, and the receptors found on different immune cell type. These actions of CUR greatly affect the innate and adaptive arms of immunity especially in the pathological conditions [34]. The peripheral analgesic and anti-ulcer effects were shown in this study following all dose levels of CUR. Surprisingly, no significant anti-inflammatory activity was observed. The analgesic activity of CUR is consistent with the previous findings of suppression of nociception in both tail flick and acetic acid-induced writhing tests following intraperitoneal injection of CUR extract [35]. Tuorkey and Karolin (2009) [36] demonstrated the anti-ulcer activity of curcumin that was displayed by attenuating the different ulcerative effectors including gastric acid hyper-secretion, total peroxides, myeloperoxidase (MPO) activity, IL-6, and apoptotic incidence.

## Conclusion

The results suggest that plants used in Thai traditional medicine for the treatment of various ailments may provide reservoirs of promising candidate chemotherapeutics for the treatment of CCA. In addition, results from pharmacological activity tests rationalize the ethnomedical use of this plant in a number of ailments. These plants have multiple pharmacological actions on various human physiological systems that would support the treatment of chronic disease like cancer. Moreover, the use of herbal medicines is safe compared with synthetic drugs. Further studies are required to determine the molecular mechanisms and active ingredients of all test materials particularly AL, which are responsible for their anti-CCA activities.

## Methods

### Chemicals and reagents

Commercial grade ethanol and acetic acid were purchased from RCI Labscan Co. Ltd. (Pathumwan, BKK, THA). The cell culture medium RPMI, fetal bovine serum (FBS), dimethylsulfoxide (DMSO), and the antibiotics-antimycotics were purchased from Gibco BRL Life Technologies (Grand Island, NY, USA). Curcumin (CUR) compound, 5-fluorouracil (5-FU), propanolol, aspirin, indomethacin, omeprazole, paracetamol, brewer's yeast, λ-carrageenan, and Tween-80 were purchased from Sigma-Aldrich Inc. (St. Louis, MO, USA). Adrenaline injection was purchased from the Government Pharmaceutical Organization of Thailand (GPO).

### Preparation of plant extracts

Plant materials were collected from various parts of Thailand and some were purchased from city markets. Authentication of plant materials were carried out at the herbarium of the Department of Forestry, Bangkok, Thailand, where the herbarium vouchers have been kept. A duplicate set was also deposited in the herbarium of Southern Center of Thai Medicinal Plants at the Faculty of Pharmaceutical Sciences, Prince of Songkhla University, Songkhla, Thailand. Preparation of the ethanolic extracts of all plant materials were performed according to the previously described method [3]. All extracts were standardized using high performance liquid chromatography to examine the amounts of active ingredients. The ethanolic extract yields of ZO, AL, PC, and PPF were 4.3, 16.89, 10.89, and 18.66%. Quantitative analysis of marker compounds of the ethanolic extracts of ZO (6-gingerol), AL (β-eudesmol), PC (piperine), and PPF (acetoxychavicol acetate) revealed 6.18, 6.64, 7.0, and 10.45% of the total crude extract, respectively.

### Cell line and culture

The CCA cell line CL-6 used for *in vivo *tumor xenograft was cultured in RPMI medium supplemented with 10% heated fetal bovine serum and 100 IU/ml of anti-anti and were maintained at 37°C in a 5% CO_2 _atmosphere with 95% humidity.

### Animals

Male and female ICR and BALB/c-nude mice (6 weeks of age, weighting 20-25 g) and Wistar albino rats (6 weeks of age, weighting 120-130 g) were purchased from The National Laboratory Animal Centre of Thailand. They were housed under standard conditions and fed with a stock diet and water *ad libitum*. Approval of the study protocol was obtained from the Ethics Committee for Research in Animals, Thammasat University, Thailand.

### Evaluation of acute and subacute toxicity

Acute and subacute toxicity tests were performed in mice and rats according to the OECD guideline for chemicals with modification in order to obtain the three dose levels (maximum tolerated dose, medium dose, and low dose) of each test material which did not cause any sign of serious toxicity or death [37]. These three tolerated dose levels were further used for assessment of the anti-CCA and pharmacological activities of each test material. ICR mice and Wistar albino rats (5 males and 5 females for each group) were fed (*via *gastric gavage) with three dose levels (low, medium, and high) of the test materials (resuspended in a mixture of distilled water and Tween-80, 4:1, v:v). The maximum dose started from 5,000 mg/kg body weight. The control animals were fed with a mixture of distilled water and Tween-80. Animals were closely observed for signs of toxicity during the first 30 minutes, periodically during the first 24 hours and then daily for 14 days (acute toxicity) or 30 days (subacute toxicity). At the end of the observational period, all animals were sacrificed under ether anesthesia and vital organs (brain, heart, kidneys, liver, spleen, stomach, large and small intestine, and lungs) were removed from all animals for gross and histopathological examination.

### Assessment of anti-CCA activity in nude mouse xenograft model

**Figure 12:**
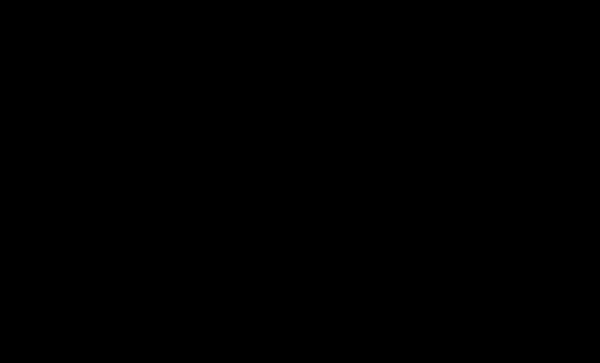
**Schematic diagram representing treatment sequence for the evaluation of anticancer activity of CUR compound and plant extracts in CCA-xenografted nude mouse model**. Cells injected = subcutaneous injection of CL-6; Treatment started = administration of treatment (CUR compound, each plant extract, vehicle or 5-FU); Treatment stopped = withdrawal of treatment (CUR compound, each plant extract, normal saline or 5-FU).

The CCA cell line CL-6 (1 × 10^6 ^cells) was used for xenografting all nude mice. Cells were removed from culture flask by trypsinization, collected in a 50 ml conical tube and centrifuged at 100 × g for 10 min. Supernatant was removed and cells were resuspended in 5 ml of complete media and cell number was counted using hemocytochamber. Cells for injection were prepared by diluting cell suspension to obtain 1 × 10^6 ^cells/200 μl normal saline (0.85% NaCl) and injected subcutaneously into the flanks of the nude mice (1 × 10^6 ^cells in 200 μl). Tumors were allowed to develop for 14 days until they reached approximately 50 mm^3 ^tumor volume. Tumor volume was measured using a caliper and body weight was recorded once every three days (Figure 12).

To evaluate the anti-CCA activity of CUR compound and each plant extract, CCA-xenografted nude mice were divided into groups of 6 males for each dose level, matched-pair according to tumor size and body weight after tumor nodules reached the volume of approximately to 50 mm^3^. All the test materials were given at the three dose levels (low, medium, and high) based on the maximum tolerated dose observed in the toxicity test. AL, ZO, and CUR were given at the doses of 1,000 (low), 3,000 (medium), and 5,000 (high) mg/kg body weight. PC was given at the doses of 100 (low), 500 (medium), and 1,000 (high) mg/kg body weight. PPF was given at the doses of 1,000 (low), 2,000 (medium), and 4,000 (high) mg/kg body weight. Each test material was fed to animals by intragastric tube (0.3 ml) daily for 30 days. The untreated and 5-FU treated control groups were given an equal volume of normal saline daily for 30 doses and 5-FU (40 μg/ml) for 14 doses, respectively. On day 0 (the day of first dose), 2, 5, 8, 11, 14, 18, 21, 24, and 28 animals were weighed on a triple-beam balance and tumor size was measured in two linear dimensions (maximum longitudinal and transverse diameters) using calipers with accuracy of 0.1 mm. The tumor volume was calculated from the formula: tumor volume = (length) (width)^2^/2.

### Autopsy and histopathological examination

For both toxicity and anticancer activity evaluation, all internal organs were removed from the animals at autopsy and observed macroscopically. Samples were fixed with 10% formalin solution. Specimens were washed in phosphate buffer three times, then dehydrated in an ascending series of ethanol for 15 min each and embedded in paraffin, followed by sectioning and staining with hematoxylin and eosin [38].

### Assessment of pharmacological activities

Each test material was administered orally to all animals (ICR mice or rats) in the form of suspension of water and 1% sodium carboxy methyl cellulose (SCMC). Animals were divided into groups of six males each. Each test material was given at the three dose levels (low, medium, and high) as described previously for the assessment of anti-CCA activities. The untreated control group received 10 ml/kg body weight of 1% SCMC while the positive control group received the reference drug for each test at an appropriate dose.

#### Locomotive activity

Rota-rod test was applied to assess the effect of CUR compound and plant extracts on motor coordination [39]. Mice were trained to remain for 3 min on the rod rotating at a speed of 25 *rpm*. On the following day, each test material, 1% SCMC (untreated control) and 4 mg/kg body weight diazepam (reference control), was administered orally to each mouse and its ability to remain on the rotating rod was assessed before and 30 min after the oral administration. The fall-off time (sec) from the rod was noted for each animal.

#### Antihypertensive activity

Rats were injected intraperitoneally with 0.1 ml of adrenaline using a 1-ml disposable syringe for five consecutive days to induce hypertension [40]. Test materials, reference drug propanolol, and 1% SCMC were given by oral administration for seven days. Blood pressure, heart rate, and mean arterial pressure were measured using CODA tail-cuff recorder (Kent Scientific, Torrington, CON, USA) machine in three steps, *i.e*., before treatment, one day after the last dose of adrenaline, and one day after the last dose of test materials or propanolol or 1% SCMC.

#### Analgesic activity

The analgesic activity of the test materials were evaluated using hot plate test [41] and writing test [42] for central and peripheral analgesic activity, respectively.

For the hot plate test, mice were placed to the beaker (on a hot plate) maintained at 55°C ± 1°C in order to obtain their response to electrical heat-induced nociceptive stimulus. Latency of analgesic response characterized by manifestation of signs of acute discomfort, *e.g*., licking of a hind limb or jumping was observed. Starting at thirty minutes after oral administration of each test material or 1% SCMC (untreated control), the analgesic response was measured at 15, 30, 45, and 60 min period. For the reference control group, morphine sulfate at a dose of 5 mg/kg body weight was injected subcutaneously into each mouse and the analgesic response was measured. Only mice that remained on the rod during the 15-45 minutes were used for initial screening and the cut-off time of 15 sec was used to evaluate analgesic activity of each test material.

For the assessment of writhing behavior, the test materials, the reference analgesic drug aspirin (200 mg/kg) or vehicle control (1% SMC) were orally administered to all mice 30 min before the intraperitoneal injection of 0.6% acetic acid (10 mg/kg body weight). The number of writhings and stretchings were counted over a 20 min period.

#### Anti-inflammatory activity

Carrageenan-induced paw edema was used to investigate the anti-inflammatory activity of the test materials [43]. The initial right hind paw volume of the rats was measured using a plethysmometer (Ugo Basile, Comerio, VA, Italy) and then 0.1 ml of 1% (w/v) λ-carrageenan was subcutaneously injected into the subplantar region of the right hind paw. The volume of the right hind paw was measured at 1, 2, 3, 4, and 5 hr after carrageenan injection. Test materials, vehicle or reference drug indomethacin (10 mg/kg body weight) were orally administered 30 min before carrageenan injection.

#### Anti-ulcer activity

The rats were fasted for 48 hours but were allowed free access to drinking water up to 2 hours before the experiment. Gastric ulcer was induced by orogastric intubation of absolute ethanol according to the method of De Pasquale et al. (1995) with modification [44]. The reference control group received an oral dose of 20 mg/kg body weight omeprazole in 1% SCMC (in volume of 5 ml/kg body weight). One hour after pre-treatment with test materials or omeprazole or 1% SCMC, all groups of rats were gavaged with absolute ethanol to induce gastric ulcer. The rats were euthanized by cervical dislocation 60 min later using diethyl ether and their stomachs were immediately excised. Ulcers found in the gastric mucosa appeared as elongated bands of hemorrhagic lesions parallel to the long axis of the stomach. Each specimen of gastric mucosa was thus examined for damage. The length (mm) and width (mm) of the ulcer on the gastric mucosa were measured under a microscope. The size of each ulcer lesion was measured and the sum of the areas of all lesions for each stomach was applied in the calculation of the ulcer area (UA), wherein the sum of small squares × 4 × 1.8 is equal to UA mm^2 ^as described previously by Kauffman and Grossman (1978) with modification [45]. The inhibition percentage (I%) was calculated as follow:

$$\left(\text{I}\%\right)=\left[\left(\text{U}{\text{A}}_{\text{control}}-\text{ U}{\text{A}}_{\text{treated}}\right)/\text{U}{\text{A}}_{\text{control}}\right]\times 100\%.$$

#### Antipyretic activity

The antipyretic activity of the test materials was evaluated using Brewer's yeast-induced pyrexia in rats [44]. Fever was induced in all rats by subcutaneous injection of 20 ml/kg boy weight of 20% aqueous suspension of Brewer's yeast in normal saline. All test materials, vehicle control (1% SCMC), and reference drug paracetamol (150 mg/kg body weight) were administered orally 17 hours after the induction of fever. Rectal temperature of each rat was recorded using thermal digital thermometer at 1, 2, 3, 4, 5, and 6 hr after the administration of the test materials, 1% SCMC, and reference drug.

### Statistical analysis

All quantitative variables were presented as mean ± SEM. Comparison of all quantitative variables between the groups treated with test materials or reference drugs with the untreated control group was performed using ANOVA, followed by *post hoc *scheffe test. Comparison of the paired quantitative variables with baseline data after treatment was performed by paired *t*-test. Statistical significance level was set at α = 0.05 for all tests.

## Competing interests

The authors declare that they have no competing interests.

## Authors' contributions

TP conducted all the experimental studies and data analysis. VE participated in animals' recruitment. KN, PP, PK, and VT participated in the design of the study. AI prepared plant materials and crude ethanolic extracts. KN finalized the manuscript. All authors read and approved the final manuscript.

## Pre-publication history

The pre-publication history for this paper can be accessed here:

http://www.biomedcentral.com/1472-6882/12/23/prepub
